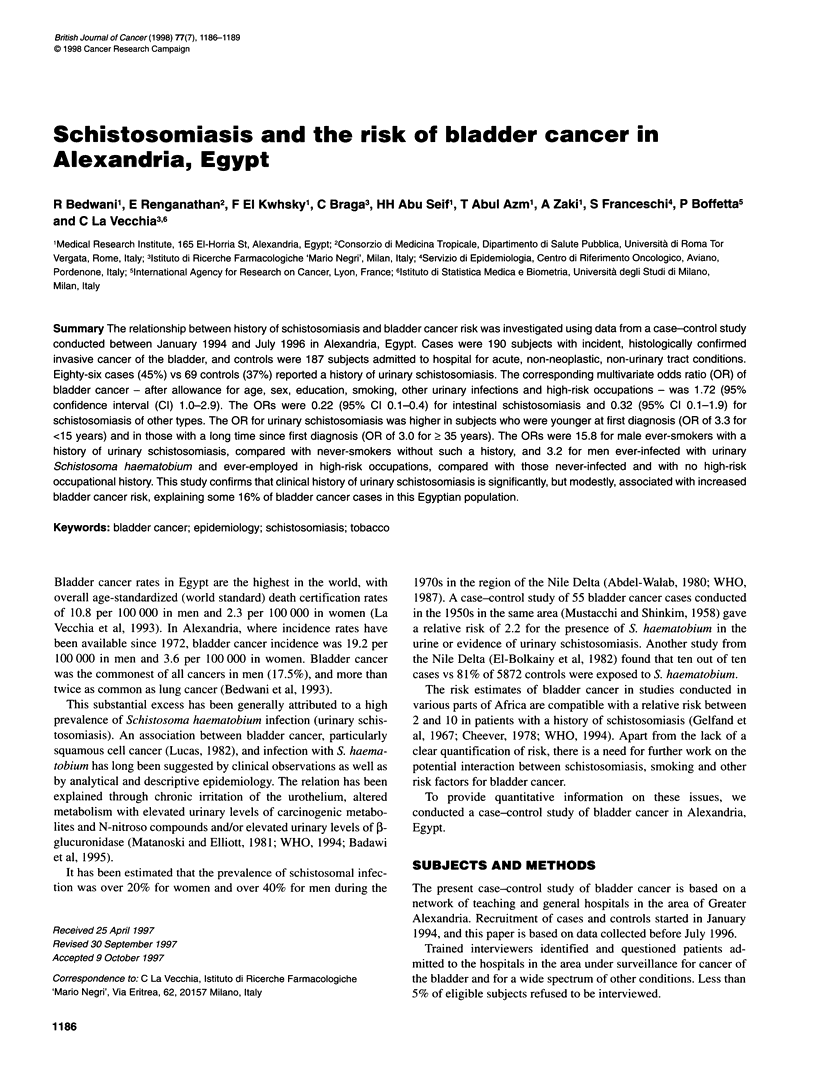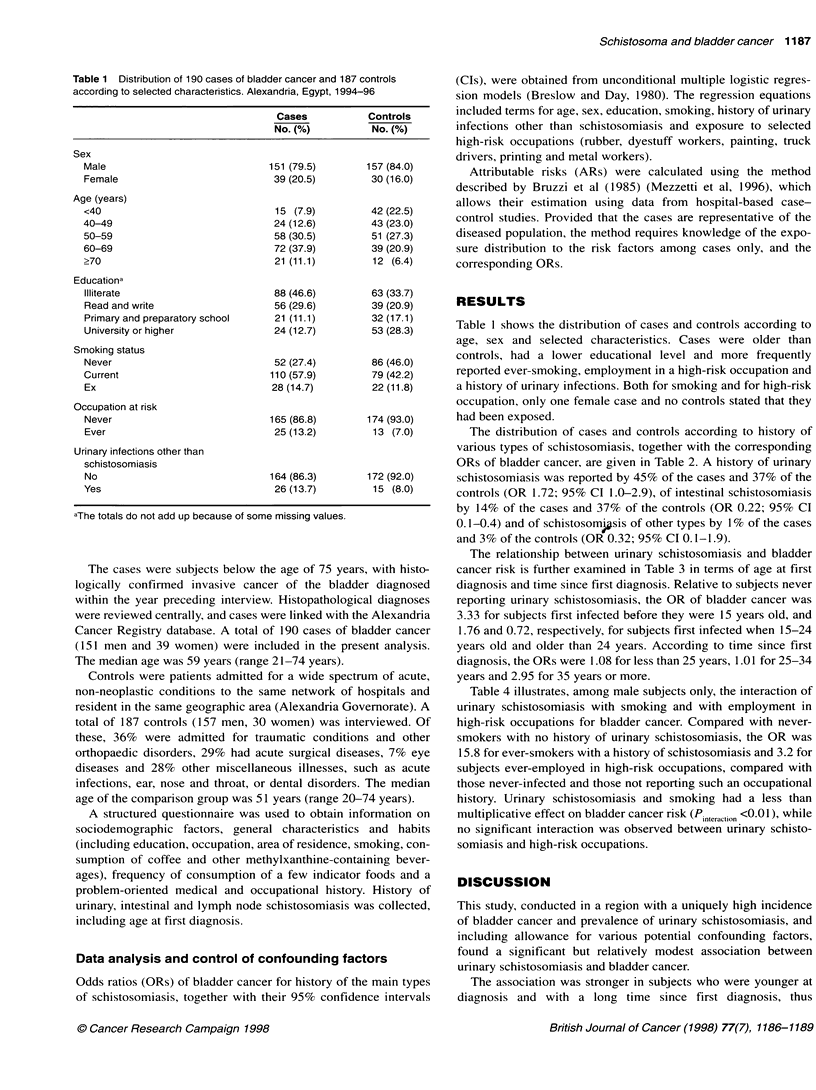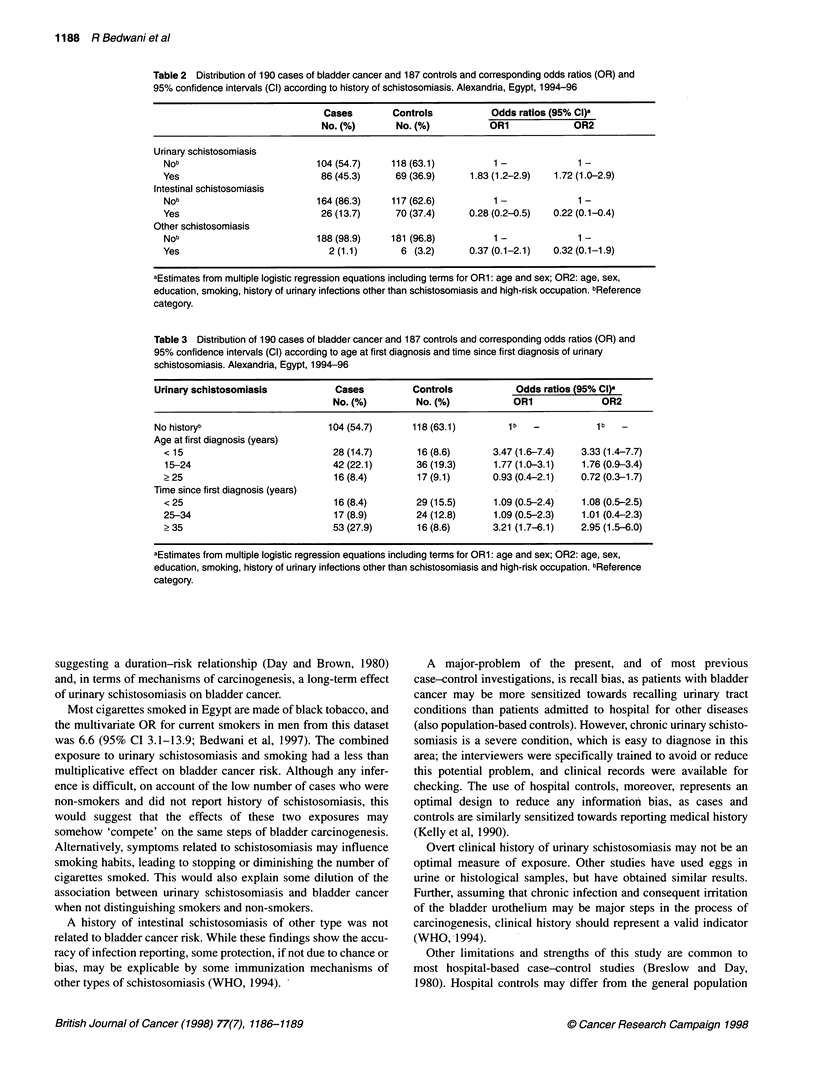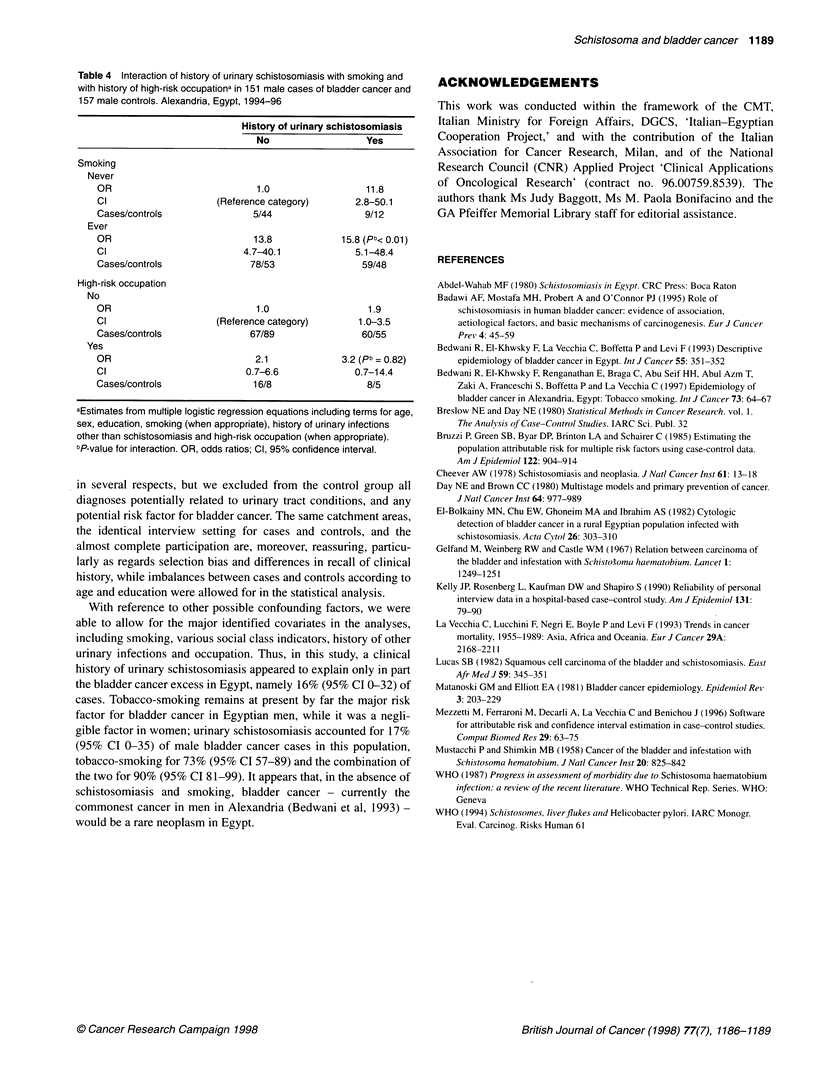# Schistosomiasis and the risk of bladder cancer in Alexandria, Egypt.

**DOI:** 10.1038/bjc.1998.197

**Published:** 1998-04

**Authors:** R. Bedwani, E. Renganathan, F. El Kwhsky, C. Braga, H. H. Abu Seif, T. Abul Azm, A. Zaki, S. Franceschi, P. Boffetta, C. La Vecchia

**Affiliations:** Medical Research Institute, Alexandria, Egypt.

## Abstract

The relationship between history of schistosomiasis and bladder cancer risk was investigated using data from a case-control study conducted between January 1994 and July 1996 in Alexandria, Egypt. Cases were 190 subjects with incident, histologically confirmed invasive cancer of the bladder, and controls were 187 subjects admitted to hospital for acute, non-neoplastic, non-urinary tract conditions. Eighty-six cases (45%) vs 69 controls (37%) reported a history of urinary schistosomiasis. The corresponding multivariate odds ratio (OR) of bladder cancer -- after allowance for age, sex, education, smoking, other urinary infections and high-risk occupations -- was 1.72 (95% confidence interval (CI) 1.0-2.9). The ORs were 0.22 (95% CI 0.1-0.4) for intestinal schistosomiasis and 0.32 (95% CI 0.1-1.9) for schistosomiasis of other types. The OR for urinary schistosomiasis was higher in subjects who were younger at first diagnosis (OR of 3.3 for <15 years) and in those with a long time since first diagnosis (OR of 3.0 for > or = 35 years). The ORs were 15.8 for male ever-smokers with a history of urinary schistosomiasis, compared with never-smokers without such a history, and 3.2 for men ever-infected with urinary Schistosoma haematobium and ever-employed in high-risk occupations, compared with those never-infected and with no high-risk occupational history. This study confirms that clinical history of urinary schistosomiasis is significantly, but modestly, associated with increased bladder cancer risk, explaining some 16% of bladder cancer cases in this Egyptian population.


British Journal of Cancer (1998) 77(7), 1186–1189
© 1998 Cancer Research Campaign

### Schistosomiasis and the risk of bladder cancer in Alexandria, Egypt

**R Bedwani[1], E Renganathan[2], F El Kwhsky[1], C Braga[3], HH Abu Seif[1], T Abul Azm[1], A Zaki[1], S Franceschi[4], P Boffetta[5] and C La Vecchia[3,6]**

[1]Medical Research Institute, 165 El-Horria St, Alexandria, Egypt; [2]Consorzio di Medicina Tropicale, Dipartimento di Salute Pubblica, Università di Roma Tor Vergata, Rome, Italy; [3]Istituto di Ricerche Farmacologiche 'Mario Negri', Milan, Italy; [4]Servizio di Epidemiologia, Centro di Riferimento Oncologico, Aviano, Pordenone, Italy; [5]International Agency for Research on Cancer, Lyon, France; [6]Istituto di Statistica Medica e Biometria, Università degli Studi di Milano, Milan, Italy

**Summary** The relationship between history of schistosomiasis and bladder cancer risk was investigated using data from a case–control study conducted between January 1994 and July 1996 in Alexandria, Egypt. Cases were 190 subjects with incident, histologically confirmed invasive cancer of the bladder, and controls were 187 subjects admitted to hospital for acute, non-neoplastic, non-urinary tract conditions. Eighty-six cases (45%) vs 69 controls (37%) reported a history of urinary schistosomiasis. The corresponding multivariate odds ratio (OR) of bladder cancer – after allowance for age, sex, education, smoking, other urinary infections and high-risk occupations – was 1.72 (95% confidence interval (CI) 1.0–2.9). The ORs were 0.22 (95% CI 0.1–0.4) for intestinal schistosomiasis and 0.32 (95% CI 0.1–1.9) for schistosomiasis of other types. The OR for urinary schistosomiasis was higher in subjects who were younger at first diagnosis (OR of 3.3 for <15 years) and in those with a long time since first diagnosis (OR of 3.0 for ≥ 35 years). The ORs were 15.8 for male ever-smokers with a history of urinary schistosomiasis, compared with never-smokers without such a history, and 3.2 for men ever-infected with urinary *Schistosoma haematobium* and ever-employed in high-risk occupations, compared with those never-infected and with no high-risk occupational history. This study confirms that clinical history of urinary schistosomiasis is significantly, but modestly, associated with increased bladder cancer risk, explaining some 16% of bladder cancer cases in this Egyptian population.

**Keywords:** bladder cancer; epidemiology; schistosomiasis; tobacco

Bladder cancer rates in Egypt are the highest in the world, with overall age-standardized (world standard) death certification rates of 10.8 per 100 000 in men and 2.3 per 100 000 in women (La Vecchia et al, 1993). In Alexandria, where incidence rates have been available since 1972, bladder cancer incidence was 19.2 per 100 000 in men and 3.6 per 100 000 in women. Bladder cancer was the commonest of all cancers in men (17.5%), and more than twice as common as lung cancer (Bedwani et al, 1993).

This substantial excess has been generally attributed to a high prevalence of *Schistosoma haematobium* infection (urinary schistosomiasis). An association between bladder cancer, particularly squamous cell cancer (Lucas, 1982), and infection with *S. haematobium* has long been suggested by clinical observations as well as by analytical and descriptive epidemiology. The relation has been explained through chronic irritation of the urothelium, altered metabolism with elevated urinary levels of carcinogenic metabolites and N-nitroso compounds and/or elevated urinary levels of β-glucuronidase (Matanoski and Elliott, 1981; WHO, 1994; Badawi et al, 1995).

It has been estimated that the prevalence of schistosomal infection was over 20% for women and over 40% for men during the 1970s in the region of the Nile Delta (Abdel-Walab, 1980; WHO, 1987). A case–control study of 55 bladder cancer cases conducted in the 1950s in the same area (Mustacchi and Shinkim, 1958) gave a relative risk of 2.2 for the presence of *S. haematobium* in the urine or evidence of urinary schistosomiasis. Another study from the Nile Delta (El-Bolkainy et al, 1982) found that ten out of ten cases vs 81% of 5872 controls were exposed to *S. haematobium*.

The risk estimates of bladder cancer in studies conducted in various parts of Africa are compatible with a relative risk between 2 and 10 in patients with a history of schistosomiasis (Gelfand et al, 1967; Cheever, 1978; WHO, 1994). Apart from the lack of a clear quantification of risk, there is a need for further work on the potential interaction between schistosomiasis, smoking and other risk factors for bladder cancer.

To provide quantitative information on these issues, we conducted a case–control study of bladder cancer in Alexandria, Egypt.

### SUBJECTS AND METHODS

The present case–control study of bladder cancer is based on a network of teaching and general hospitals in the area of Greater Alexandria. Recruitment of cases and controls started in January 1994, and this paper is based on data collected before July 1996.

Trained interviewers identified and questioned patients admitted to the hospitals in the area under surveillance for cancer of the bladder and for a wide spectrum of other conditions. Less than 5% of eligible subjects refused to be interviewed.

*Received 25 April 1997*
*Revised 30 September 1997*
*Accepted 9 October 1997*

*Correspondence to:* C La Vecchia, Istituto di Ricerche Farmacologiche 'Mario Negri', Via Eritrea, 62, 20157 Milano, Italy

**Table 1** Distribution of 190 cases of bladder cancer and 187 controls according to selected characteristics. Alexandria, Egypt, 1994–96

| | Cases<br>No. (%) | Controls<br>No. (%) |
|---|---|---|
| Sex | | |
| Male | 151 (79.5) | 157 (84.0) |
| Female | 39 (20.5) | 30 (16.0) |
| Age (years) | | |
| <40 | 15 (7.9) | 42 (22.5) |
| 40–49 | 24 (12.6) | 43 (23.0) |
| 50–59 | 58 (30.5) | 51 (27.3) |
| 60–69 | 72 (37.9) | 39 (20.9) |
| ≥70 | 21 (11.1) | 12 (6.4) |
| Education[a] | | |
| Illiterate | 88 (46.6) | 63 (33.7) |
| Read and write | 56 (29.6) | 39 (20.9) |
| Primary and preparatory school | 21 (11.1) | 32 (17.1) |
| University or higher | 24 (12.7) | 53 (28.3) |
| Smoking status | | |
| Never | 52 (27.4) | 86 (46.0) |
| Current | 110 (57.9) | 79 (42.2) |
| Ex | 28 (14.7) | 22 (11.8) |
| Occupation at risk | | |
| Never | 165 (86.8) | 174 (93.0) |
| Ever | 25 (13.2) | 13 (7.0) |
| Urinary infections other than schistosomiasis | | |
| No | 164 (86.3) | 172 (92.0) |
| Yes | 26 (13.7) | 15 (8.0) |

[a]The totals do not add up because of some missing values.

The cases were subjects below the age of 75 years, with histologically confirmed invasive cancer of the bladder diagnosed within the year preceding interview. Histopathological diagnoses were reviewed centrally, and cases were linked with the Alexandria Cancer Registry database. A total of 190 cases of bladder cancer (151 men and 39 women) were included in the present analysis. The median age was 59 years (range 21–74 years).

Controls were patients admitted for a wide spectrum of acute, non-neoplastic conditions to the same network of hospitals and resident in the same geographic area (Alexandria Governorate). A total of 187 controls (157 men, 30 women) was interviewed. Of these, 36% were admitted for traumatic conditions and other orthopaedic disorders, 29% had acute surgical diseases, 7% eye diseases and 28% other miscellaneous illnesses, such as acute infections, ear, nose and throat, or dental disorders. The median age of the comparison group was 51 years (range 20–74 years).

A structured questionnaire was used to obtain information on sociodemographic factors, general characteristics and habits (including education, occupation, area of residence, smoking, consumption of coffee and other methylxanthine-containing beverages), frequency of consumption of a few indicator foods and a problem-oriented medical and occupational history. History of urinary, intestinal and lymph node schistosomiasis was collected, including age at first diagnosis.

### Data analysis and control of confounding factors

Odds ratios (ORs) of bladder cancer for history of the main types of schistosomiasis, together with their 95% confidence intervals (CIs), were obtained from unconditional multiple logistic regression models (Breslow and Day, 1980). The regression equations included terms for age, sex, education, smoking, history of urinary infections other than schistosomiasis and exposure to selected high-risk occupations (rubber, dyestuff workers, painting, truck drivers, printing and metal workers).

Attributable risks (ARs) were calculated using the method described by Bruzzi et al (1985) (Mezzetti et al, 1996), which allows their estimation using data from hospital-based case–control studies. Provided that the cases are representative of the diseased population, the method requires knowledge of the exposure distribution to the risk factors among cases only, and the corresponding ORs.

### RESULTS

Table 1 shows the distribution of cases and controls according to age, sex and selected characteristics. Cases were older than controls, had a lower educational level and more frequently reported ever-smoking, employment in a high-risk occupation and a history of urinary infections. Both for smoking and for high-risk occupation, only one female case and no controls stated that they had been exposed.

The distribution of cases and controls according to history of various types of schistosomiasis, together with the corresponding ORs of bladder cancer, are given in Table 2. A history of urinary schistosomiasis was reported by 45% of the cases and 37% of the controls (OR 1.72; 95% CI 1.0–2.9), of intestinal schistosomiasis by 14% of the cases and 37% of the controls (OR 0.22; 95% CI 0.1–0.4) and of schistosomiasis of other types by 1% of the cases and 3% of the controls (OR 0.32; 95% CI 0.1–1.9).

The relationship between urinary schistosomiasis and bladder cancer risk is further examined in Table 3 in terms of age at first diagnosis and time since first diagnosis. Relative to subjects never reporting urinary schistosomiasis, the OR of bladder cancer was 3.33 for subjects first infected before they were 15 years old, and 1.76 and 0.72, respectively, for subjects first infected when 15–24 years old and older than 24 years. According to time since first diagnosis, the ORs were 1.08 for less than 25 years, 1.01 for 25–34 years and 2.95 for 35 years or more.

Table 4 illustrates, among male subjects only, the interaction of urinary schistosomiasis with smoking and with employment in high-risk occupations for bladder cancer. Compared with never-smokers with no history of urinary schistosomiasis, the OR was 15.8 for ever-smokers with a history of schistosomiasis and 3.2 for subjects ever-employed in high-risk occupations, compared with those never-infected and those not reporting such an occupational history. Urinary schistosomiasis and smoking had a less than multiplicative effect on bladder cancer risk ($P_{interaction}$ <0.01), while no significant interaction was observed between urinary schistosomiasis and high-risk occupations.

### DISCUSSION

This study, conducted in a region with a uniquely high incidence of bladder cancer and prevalence of urinary schistosomiasis, and including allowance for various potential confounding factors, found a significant but relatively modest association between urinary schistosomiasis and bladder cancer.

The association was stronger in subjects who were younger at diagnosis and with a long time since first diagnosis, thus

© Cancer Research Campaign 1998

**Table 2** Distribution of 190 cases of bladder cancer and 187 controls and corresponding odds ratios (OR) and 95% confidence intervals (CI) according to history of schistosomiasis. Alexandria, Egypt, 1994–96

| | Cases No. (%) | Controls No. (%) | Odds ratios (95% CI)[a] OR1 | OR2 |
|---|---|---|---|---|
| Urinary schistosomiasis | | | | |
| No[b] | 104 (54.7) | 118 (63.1) | 1 – | 1 – |
| Yes | 86 (45.3) | 69 (36.9) | 1.83 (1.2–2.9) | 1.72 (1.0–2.9) |
| Intestinal schistosomiasis | | | | |
| No[b] | 164 (86.3) | 117 (62.6) | 1 – | 1 – |
| Yes | 26 (13.7) | 70 (37.4) | 0.28 (0.2–0.5) | 0.22 (0.1–0.4) |
| Other schistosomiasis | | | | |
| No[b] | 188 (98.9) | 181 (96.8) | 1 – | 1 – |
| Yes | 2 (1.1) | 6 (3.2) | 0.37 (0.1–2.1) | 0.32 (0.1–1.9) |

[a]Estimates from multiple logistic regression equations including terms for OR1: age and sex; OR2: age, sex, education, smoking, history of urinary infections other than schistosomiasis and high-risk occupation. [b]Reference category.

**Table 3** Distribution of 190 cases of bladder cancer and 187 controls and corresponding odds ratios (OR) and 95% confidence intervals (CI) according to age at first diagnosis and time since first diagnosis of urinary schistosomiasis. Alexandria, Egypt, 1994–96

| Urinary schistosomiasis | Cases No. (%) | Controls No. (%) | Odds ratios (95% CI)[a] OR1 | OR2 |
|---|---|---|---|---|
| No history[b] | 104 (54.7) | 118 (63.1) | 1[b] – | 1[b] – |
| Age at first diagnosis (years) | | | | |
| < 15 | 28 (14.7) | 16 (8.6) | 3.47 (1.6–7.4) | 3.33 (1.4–7.7) |
| 15–24 | 42 (22.1) | 36 (19.3) | 1.77 (1.0–3.1) | 1.76 (0.9–3.4) |
| ≥ 25 | 16 (8.4) | 17 (9.1) | 0.93 (0.4–2.1) | 0.72 (0.3–1.7) |
| Time since first diagnosis (years) | | | | |
| < 25 | 16 (8.4) | 29 (15.5) | 1.09 (0.5–2.4) | 1.08 (0.5–2.5) |
| 25–34 | 17 (8.9) | 24 (12.8) | 1.09 (0.5–2.3) | 1.01 (0.4–2.3) |
| ≥ 35 | 53 (27.9) | 16 (8.6) | 3.21 (1.7–6.1) | 2.95 (1.5–6.0) |

[a]Estimates from multiple logistic regression equations including terms for OR1: age and sex; OR2: age, sex, education, smoking, history of urinary infections other than schistosomiasis and high-risk occupation. [b]Reference category.

suggesting a duration–risk relationship (Day and Brown, 1980) and, in terms of mechanisms of carcinogenesis, a long-term effect of urinary schistosomiasis on bladder cancer.

Most cigarettes smoked in Egypt are made of black tobacco, and the multivariate OR for current smokers in men from this dataset was 6.6 (95% CI 3.1–13.9; Bedwani et al, 1997). The combined exposure to urinary schistosomiasis and smoking had a less than multiplicative effect on bladder cancer risk. Although any inference is difficult, on account of the low number of cases who were non-smokers and did not report history of schistosomiasis, this would suggest that the effects of these two exposures may somehow 'compete' on the same steps of bladder carcinogenesis. Alternatively, symptoms related to schistosomiasis may influence smoking habits, leading to stopping or diminishing the number of cigarettes smoked. This would also explain some dilution of the association between urinary schistosomiasis and bladder cancer when not distinguishing smokers and non-smokers.

A history of intestinal schistosomiasis of other type was not related to bladder cancer risk. While these findings show the accuracy of infection reporting, some protection, if not due to chance or bias, may be explicable by some immunization mechanisms of other types of schistosomiasis (WHO, 1994).

A major-problem of the present, and of most previous case–control investigations, is recall bias, as patients with bladder cancer may be more sensitized towards recalling urinary tract conditions than patients admitted to hospital for other diseases (also population-based controls). However, chronic urinary schistosomiasis is a severe condition, which is easy to diagnose in this area; the interviewers were specifically trained to avoid or reduce this potential problem, and clinical records were available for checking. The use of hospital controls, moreover, represents an optimal design to reduce any information bias, as cases and controls are similarly sensitized towards reporting medical history (Kelly et al, 1990).

Overt clinical history of urinary schistosomiasis may not be an optimal measure of exposure. Other studies have used eggs in urine or histological samples, but have obtained similar results. Further, assuming that chronic infection and consequent irritation of the bladder urothelium may be major steps in the process of carcinogenesis, clinical history should represent a valid indicator (WHO, 1994).

Other limitations and strengths of this study are common to most hospital-based case–control studies (Breslow and Day, 1980). Hospital controls may differ from the general population

© Cancer Research Campaign 1998

**Table 4** Interaction of history of urinary schistosomiasis with smoking and with history of high-risk occupation[a] in 151 male cases of bladder cancer and 157 male controls. Alexandria, Egypt, 1994–96

| | History of urinary schistosomiasis | |
|---|---|---|
| | **No** | **Yes** |
| Smoking | | |
| Never | | |
| OR | 1.0 | 11.8 |
| CI | (Reference category) | 2.8–50.1 |
| Cases/controls | 5/44 | 9/12 |
| Ever | | |
| OR | 13.8 | 15.8 ($P^b$< 0.01) |
| CI | 4.7–40.1 | 5.1–48.4 |
| Cases/controls | 78/53 | 59/48 |
| High-risk occupation | | |
| No | | |
| OR | 1.0 | 1.9 |
| CI | (Reference category) | 1.0–3.5 |
| Cases/controls | 67/89 | 60/55 |
| Yes | | |
| OR | 2.1 | 3.2 ($P^b$ = 0.82) |
| CI | 0.7–6.6 | 0.7–14.4 |
| Cases/controls | 16/8 | 8/5 |

[a]Estimates from multiple logistic regression equations including terms for age, sex, education, smoking (when appropriate), history of urinary infections other than schistosomiasis and high-risk occupation (when appropriate). [b]*P*-value for interaction. OR, odds ratios; CI, 95% confidence interval.

in several respects, but we excluded from the control group all diagnoses potentially related to urinary tract conditions, and any potential risk factor for bladder cancer. The same catchment areas, the identical interview setting for cases and controls, and the almost complete participation are, moreover, reassuring, particularly as regards selection bias and differences in recall of clinical history, while imbalances between cases and controls according to age and education were allowed for in the statistical analysis.

With reference to other possible confounding factors, we were able to allow for the major identified covariates in the analyses, including smoking, various social class indicators, history of other urinary infections and occupation. Thus, in this study, a clinical history of urinary schistosomiasis appeared to explain only in part the bladder cancer excess in Egypt, namely 16% (95% CI 0–32) of cases. Tobacco-smoking remains at present by far the major risk factor for bladder cancer in Egyptian men, while it was a negligible factor in women; urinary schistosomiasis accounted for 17% (95% CI 0–35) of male bladder cancer cases in this population, tobacco-smoking for 73% (95% CI 57–89) and the combination of the two for 90% (95% CI 81–99). It appears that, in the absence of schistosomiasis and smoking, bladder cancer – currently the commonest cancer in men in Alexandria (Bedwani et al, 1993) – would be a rare neoplasm in Egypt.

### ACKNOWLEDGEMENTS

This work was conducted within the framework of the CMT, Italian Ministry for Foreign Affairs, DGCS, 'Italian–Egyptian Cooperation Project,' and with the contribution of the Italian Association for Cancer Research, Milan, and of the National Research Council (CNR) Applied Project 'Clinical Applications of Oncological Research' (contract no. 96.00759.8539). The authors thank Ms Judy Baggott, Ms M. Paola Bonifacino and the GA Pfeiffer Memorial Library staff for editorial assistance.

### REFERENCES

Abdel-Wahab MF (1980) *Schistosomiasis in Egypt*. CRC Press: Boca Raton

Badawi AF, Mostafa MH, Probert A and O'Connor PJ (1995) Role of schistosomiasis in human bladder cancer: evidence of association, aetiological factors, and basic mechanisms of carcinogenesis. *Eur J Cancer Prev* **4**: 45–59

Bedwani R, El-Khwsky F, La Vecchia C, Boffetta P and Levi F (1993) Descriptive epidemiology of bladder cancer in Egypt. *Int J Cancer* **55**: 351–352

Bedwani R, El-Khwsky F, Renganathan E, Braga C, Abu Seif HH, Abul Azm T, Zaki A, Franceschi S, Boffetta P and La Vecchia C (1997) Epidemiology of bladder cancer in Alexandria, Egypt: Tobacco smoking. *Int J Cancer* **73**: 64–67

Breslow NE and Day NE (1980) *Statistical Methods in Cancer Research*. vol. 1. *The Analysis of Case-Control Studies*. IARC Sci. Publ. 32

Bruzzi P, Green SB, Byar DP, Brinton LA and Schairer C (1985) Estimating the population attributable risk for multiple risk factors using case-control data. *Am J Epidemiol* **122**: 904–914

Cheever AW (1978) Schistosomiasis and neoplasia. *J Natl Cancer Inst* **61**: 13–18

Day NE and Brown CC (1980) Multistage models and primary prevention of cancer. *J Natl Cancer Inst* **64**: 977–989

El-Bolkainy MN, Chu EW, Ghoneim MA and Ibrahim AS (1982) Cytologic detection of bladder cancer in a rural Egyptian population infected with schistosomiasis. *Acta Cytol* **26**: 303–310

Gelfand M, Weinberg RW and Castle WM (1967) Relation between carcinoma of the bladder and infestation with *Schistosoma haematobium*. *Lancet* **1**: 1249–1251

Kelly JP, Rosenberg L, Kaufman DW and Shapiro S (1990) Reliability of personal interview data in a hospital-based case–control study. *Am J Epidemiol* **131**: 79–90

La Vecchia C, Lucchini F, Negri E, Boyle P and Levi F (1993) Trends in cancer mortality, 1955–1989: Asia, Africa and Oceania. *Eur J Cancer* **29A**: 2168–2211

Lucas SB (1982) Squamous cell carcinoma of the bladder and schistosomiasis. *East Afr Med J* **59**: 345–351

Matanoski GM and Elliott EA (1981) Bladder cancer epidemiology. *Epidemiol Rev* **3**: 203–229

Mezzetti M, Ferraroni M, Decarli A, La Vecchia C and Benichou J (1996) Software for attributable risk and confidence interval estimation in case–control studies. *Comput Biomed Res* **29**: 63–75

Mustacchi P and Shimkin MB (1958) Cancer of the bladder and infestation with *Schistosoma hematobium*. *J Natl Cancer Inst* **20**: 825–842

WHO (1987) *Progress in assessment of morbidity due to* Schistosoma haematobium *infection: a review of the recent literature*. WHO Technical Rep. Series. WHO: Geneva

WHO (1994) *Schistosomes, liver flukes and* Helicobacter pylori. IARC Monogr. Eval. Carcinog. Risks Human 61

© Cancer Research Campaign 1998